# Psychometric properties and parental reported utility of the 19-item ‘About My Child’ (AMC-19) measure

**DOI:** 10.1186/s12887-018-1147-2

**Published:** 2018-05-25

**Authors:** Uzma Williams, Peter Rosenbaum, Jan Willem Gorter, Dayle McCauley, Roman Gulko

**Affiliations:** School of Rehabilitation Sciences, CanChild, McMaster University, Institute for Applied Health Sciences, 1200 Main Street West, Hamilton, ON L8N 3Z5 Canada

**Keywords:** About My Child, Childhood, Complexity, Development, Disability, International Classification of Functioning, Disability and Health, Reliability

## Abstract

**Background:**

‘About My Child’ 19-item version (AMC-19) is a parent-report measure developed to assess the complexity of a child’s life due to biological, psychological, social and environmental issues, that can be completed in approximately 5 min. AMC measures two dimensions of complexity: parental concerns and impact on the child. This paper examines the psychometric properties and parent-reported utility of the AMC-19 for children with disabilities or special health care needs.

**Method:**

Data were gathered from two Canadian studies at *CanChild*: the ‘AMC-19 Pilot’ study and the ‘Service Utilization and Outcomes (SUO)’ study. The AMC-19 Pilot study data allowed us to explore internal consistency and test-retest reliability, as well as parental responses to two open-ended questions on the utility of the AMC-19. The SUO study provided data for analyses of internal consistency and scale property validation with type of diagnosis and service needs.

**Results:**

The test-retest ICC was *r* = 0.83 for concerns and *r* = 0.87 for impact. Cronbach’s alpha across both studies ranged from 0.80 to 0.90. Parents’ comments on the AMC-19’s utility indicated support for the AMC-19, in particular to identify therapy needs and goals.

**Conclusions:**

The AMC-19 demonstrates strong psychometric properties supporting it as a valuable measure for describing the level of complexity among children with disabilities. We recommend using the AMC-19 in health services research and clinical settings to build dialogue between family and therapists due to its utility reported by parents.

## Background

Understanding and assessing childhood impairment and disability remain significant public health concerns. More than 93 million (or 5.1%) children and adolescents below age 15 live with a childhood disability around the world [1]. Children with disabilities are often diagnosed with multiple ‘primary’ health conditions, while associated impairments (‘co-morbidities’) vary greatly in both scope and severity, resulting in a need for a wide variety of additional services and supports [2]. Children with chronic impairments often lead complex lives in terms of their health care needs, and this reality often impacts their quality of life and that of their families [3]. In order to provide families using rehabilitation services with optimal care as well as improved living standards and life quality, it is important to describe, measure, and assess accurately the ‘complexity’ of children with impairments or special health care needs.

Defining complexity has proved to be a troublesome task by virtue of the clinician’s dependence on contextual and subjective information. In addition, due to the lack of a clear definition, “complexity” has been erroneously conflated with other constructs such as co-morbidity and severity [4, 5]. Nonetheless, several attempts have been made to define complexity. A comprehensive description of complexity has been provided as the cumulative number of biological, psychological, social and environmental issues in a child’s life that directly (and usually negatively) impact the child’s health and care needs [5]. This notion proposes that the simple cumulative count of child health-related issues and their impact is more salient than the severity of each individual issue. Complexity is differentiated from a variety of related concepts, including disease severity, illness severity, comorbidity, functional ability, health utility, quality of life, and others [6].

It is argued that thinking about complexity in this way is a more useful indicator of functioning in daily life activities than severity of the condition(s), because the latter does not consider or aggregate the multiple areas of concern or the magnitude and cumulative impact of these concerns. One can have an objectively observed ‘functional limitation’ – some limited level of capacity to do something – and have good accommodations that allow people to ‘do’ things even if they ‘do’ them differently from what is considered typical. For example, a child may have a walking limitation. With modifications in their home and school environment, this child may not be limited in independent mobility or participation in daily life activities, so the impact of the body-level impairment is minor. On the other hand, another child with the same level of functional limitation may report a higher level of complexity and thus find difficulty engaging in daily life activities if environmental modifications do not accommodate their needs (or indeed if additional functional challenges are present). In this situation, the parents of the second child will almost certainly report a higher level of concern and complexity.

Although several instruments and methods have been designed to measure complexity in adult health, one tool that measures complexity in children with disabilities by using a parent proxy response is the About My Child (AMC) measure [6]. The AMC measures child health complexity and has been proposed as a tool to understand the cumulative functional needs of children and the priorities of their families. The AMC measure was created in the context of the International Classification of Functioning, Disability and Health (ICF) framework (see Table 1), which is a classification framework used for measuring health and disability [7].

**Table 1:** ICF Coding for the AMC Measure^a^

*Question* I am concerned about my child’s:	ICF Chapter	ICF Codes
1. Ability to move around at home, school and community	AP-4 - Mobility	d 460
2. Ability to use their hands and arms to do the things they want to do	AP-4 - Mobility	d 445, d 440
3. Ability to perform functions such as feeding/eating	AP-5 - Self-Care	d 550, d 560
4. Ability to carry out toileting	AP-5 - Self-Care	d 530
5. Ability to dress or undress self	AP-5 - Self-Care	d 540
6. Ability to sleep each night	BF-1 - Mental Functions	b 134
7. Seeing	BF-2 - Sensory Functions	b 210
8. Hearing	BF-2 - Sensory Functions	b 230
9. Ability to understand other people	BF-1 - Mental Functions, AP-3 - Communication	b 1670, d 310, d315
10. Ability to tell people what they want	BF-1 - Mental functions, AP-3 - Communication	b 1671, d 330, d 315
11. Behavior	AP-2 - General tasks and demands	d250
12. Mood	BF-1 - Mental Functions	b 152
13. Pain	BF2 - Sensory Functions	b 280
14. Ability to learn new things	BF-1 - Mental Functions, AP-1 - Learning and applying knowledge	b 163, d 130-d159
15. Ability to remember things they know	BF-1 - Mental Functions, AP - 1 Learning and applying knowledge	b 144, d 179
16. Ability to get along with other children	AP-7 - Interpersonal relationships	d 7504
17. Ability to get along with adults	AP-7 - Interpersonal relationships	d 7203,
18. Participation in activities at home	–	d 880
19. Participate in activities at school or in the community	AP-8 - Major life areas, AP-9 - Community, social and civic life	d 820, d 910

^a^The ICF encompasses four major health domains: body functions (b), body structures (s), activities and participation (d), and environmental factors (e), and is organized using alphanumeric codes. The letter within the alphanumeric code corresponds to a health domain and the numbers act as qualifiers, indicating severity. The AMC inventory was created so that each of the 19 items on the questionnaire corresponds to an ICF alphanumeric code. Table 1 summarizes the main focus of each AMC item and its relationship with its corresponding ICF code

While the AMC was developed empirically, it has been used by students and researchers at *CanChild* despite the absence of a formal measurement development process. Two versions of the AMC exist: an earlier 26-item version and a later 19-item version presented here, both available from *CanChild* [6].

While Ritzema and Lach [8] reported on the reliability and validity of the 26-item AMC measure, showing good internal consistency (Cronbach’s alpha = 0.75 to 0.85), they also indicated the need for completing test-retest for the measure, in addition to exploring clinical utility. Factor analysis was conducted on the original 26-item AMC measure (not shown) to explore the principal component loadings. Items were optimized and redundant items were removed, producing the current 19-item AMC version (hereafter referred to as the AMC-19).

The purpose of the current report is to assess the psychometric properties and ‘performance’ of the AMC-19. Assessing the capability of the AMC-19 may validate its use in health services research and advance the use of the AMC-19 in clinical applications, such as for collaboration between clinicians and parents for functional goal setting in line with family-centred care. This paper reports the test-retest reliability, scale properties, and parent reported utility of the AMC-19, using data from the Pilot study and the Service Utilization and Outcomes (‘SUO’) study [9].

## Methods

### Measures

#### AMC

The AMC-19 is designed to allow family members and caregivers to highlight the issues that are important to each individual family regarding the condition and care of their child. It does so using a checklist of the most common parental concerns (for example, ability to move around, feed/eat, toilet, understand, communicate, learn, participate in activities, etc.). Each item has a similar structure, with two parts to each question reporting two components: *concern* and *perceived impact*, both indicating complexity. *Concern* refers to the parents’ perception about issues affecting their child’s functioning, and the extent to which that concern (if present) is thought to *impact* the child’s life and function. Parents rate the *concern* questions as either “yes” or “no” for each of the items. Each item states: “I am concerned about my child’s… [e.g., “Ability to **move around** at home, school and community”]. If the parent answers “yes”, they are asked to respond to: “If yes, does this *impact* on their ability to participate in everyday activities?” ranging on a four-point response option from “not at all” to “a lot”. The first approach to score the AMC-19 counts the *number* of concerns parents have about their child. Areas of concern are reported by summing the number of issues identified by parents. Items scored “yes” receive 1 point whereas a response of “no” is scored as 0, with ‘concern’ scores ranging from 0 to 19. The second component, *impact*, reports on the parent’s perceptions of the complications in the child’s life imposed by the issues they have identified. Each item that is indicated as a concern by checking “yes” is rated on a 4-point Likert-type scale to assess the impact on the child’s functioning 1 (“not at all”), 2 (“a little”), 3 (“somewhat”), and 4 (“a lot”). Thus, if the respondent replies “yes” to the first part of an item, then the value of “yes” (1) is multiplied by the value of the response to the second part. The scores of all 19 items are then summed for a total ‘complexity’ score that can range from 0 to 76. The AMC-19 can be completed in approximately 5 min.

#### Supports and Services (SAS) questionnaire

The SAS questionnaire provides an understanding of the perceived service needs required by the child and their family [10]. The SAS is a 28-item list inventory split into two sections, with 14 child items and 14 family items. Psychometric evaluations of the SAS have not been conducted due to the dynamic nature of the measure [Jean Summers, personal communication, February 17, 2016]; however, the clinical utility and its use in a treatment outcome measurement system for children using a CTC are supported [11]. Like the AMC-19, the SAS is structured with a stem that asks “Does your child currently need…” e.g., “special equipment”. The parent can respond “Yes” or “No”. Each question then asks “If yes, how much service does he or she get?” with three response options of “none”, “some but not enough”, or “enough”*.* For the purpose of this study, the SAS was scored similarly to the AMC. The researchers of this study provided a score of 1 to “yes” and 0 to “no” and then multiplied this score by 3 (“none”), 2 (“some but not enough”), or 1 (“enough”) services received. The possible range is 0 to 78, with a higher score indicative of the family needing more services.

### Data sources for establishing psychometric properties and clinical utility

The sources of the two datasets used to assess the psychometrics of the AMC-19 are summarized below. Both studies met sample size requirements for conducting reliability analysis [12]. Ethics approval was obtained from McMaster University and Hamilton Integrated Research Ethics Board for the AMC-19 Pilot study and SUO study, respectively.

1. AMC-19 Pilot study: The AMC-19 Pilot study was part of a larger body of work conducted by *CanChild* between 2004 and 2008. During this period, *CanChild* was developing a provincial measurement system to help government assess services to determine meaningful outcomes for children and youth with special needs and their families [11]. In consultation with various stakeholders, the ICF framework [7] for this system was chosen along with several instruments that were identified to be the best specific measures to evaluate the constructs of interest [11]. The best measure of body function and structure was determined to be the AMC-19, however, at the time no information was available on its psychometric properties. To gather information about the reliability of this measure, a group of researchers worked in partnership with selected publicly-funded regional Southern Ontario Children’s Treatment Centres (CTCs) to assess test-retest reliability of the AMC-19 as part of a larger study to assess the test-retest reliability of the Craig Hospital Inventory of Environmental Factors (CHIEF) for children 2–12 years of age [13]. This pilot study used cold one-time mail-out to distribute 450 surveys to families in three CTCs in Ontario (packages were sent directly from the respective CTCs) and received data from 61 families. The initial correspondence and survey were mailed out in March 2009 and a retest survey was mailed 2 weeks after the initial questionnaires were received at *CanChild*. Of the 61 initial returned packages, 45 (73.8%) retest packages were received at *CanChild*.
2. SUO study: The SUO study recruitment and methods are described in detail in Williams’ PhD dissertation [9]. Using a cross-sectional design, the SUO study explored personal, family, and environmental factors of children using a CTC in Southern Ontario. The online survey completed by parents included demographic questions, AMC-19 measure, SAS questionnaire, as well as measures of children’s participation and family-centred practices. Seven hundred families were invited to the study and 202 fully completed the AMC-19 used in this analysis.

### Study participants

Participant demographics are summarized in Table 2. The participant groups in both studies consisted of parents of more males than females (65.6% in the AMC-19 Pilot study and 61.9% in the SUO study) with a mean of 6 years (SD = 3.0) in the AMC-19 Pilot study and 4.5 years (SD = 3.0) in the SUO study. The primary diagnoses of children varied in the AMC-19 Pilot and SUO studies, and included Autism Spectrum Disorder (ASD)-related disorders, speech difficulties, cerebral palsy (CP), Developmental Delay (DD) such as learning or motor delays, syndromes such as Down Syndrome, and children diagnosed with two or more conditions. The primary diagnosis in the AMC-19 Pilot study was DD (*n* = 17, 27.9%); in the SUO study, the communication disorder/speech delay diagnoses involved 63 participants (25.0%), and DD diagnoses involved 53 participants (21%). In the AMC-19 Pilot study, parent reported complexity scores ranged from 7 to 69 on initial completion and 2 to 65 on retest completion. Complexity scores ranged from 1 to 56 in the SUO study.

**Table 2:** Participant characteristics

Demographic	AMC Pilot Data N (%)		SUO Data N (%)	
Child’s age				
0–2 years 11 months	3	(4.9)	43	(17.1)
3–5 years 11 months	29	(47.5)	170	(67.4)
6–11 years 11 months	27	(44.3)	26	(10.3)
12 years and older	2	(3.3)	13	(5.2)
Child’s sex				
Male	40	(65.6)	156	(61.9)
Female	21	(34.4)	96	(38.1)
Child’s diagnosis				
Acquired brain injury	1	(1.6)	8	(3.2)
ASD-related disorders/PDD	7	(11.5)	26	(10.3)
Cerebral palsy	7	(11.5)	20	(8.0)
Communication disorder/speech delay	3	(4.9)	63	(25.0)
DCD	2	(3.3)	1	(0.4)
DD	17	(27.9)	53	(21.0)
Neuromuscular disease (including muscular dystrophy)	2	(3.3)	5	(2.0)
Spina bifida/hydrocephalus	2	(3.3)	3	(1.2)
Syndrome	9	(14.7)	16	(6.3)
Two or more conditions	–	–	16	(6.3)
Other	11	(18.0)	24	(9.5)
Missing	–	–	17	(6.8)
Living Arrangement				
Two-parent family	55	(90.2)	223	(88.5)
Single-parent family	6	(9.8)	24	(9.5)
Other	–	–	3	(1.2)
Missing	–	–	2	(0.8)
Parent’s age (years)				
20–34	18	(29.5)	114	(45.2)
35–49	41	(67.2)	130	(51.6)
50–64	2	(3.3)	5	(2.0)
Missing	–	–	3	(1.2)
Parent’s income (%)				
Less than 15 K?	4	(6.6)	14	(5.6)
15,000–29,999	7	(11.5)	21	(8.3)
30,000–44,999	8	(13.1)	21	(8.3)
45,000–59,999	7	(11.5)	30	(11.9)
60,000–74,999	11	(18.0)	30	(11.9)
75,000–89,999	5	(8.2)	25	(9.9)
More than 90,000	18	(29.5)	105	(41.7)
Missing	1	(1.6)	6	(2.4)
Highest level of education completed by respondent				
Some high school (grades 9–11)	2	(3.3)	11	(4.4)
Completed high school	8	(13.1)	28	(11.1)
Some college or technical training	6	(9.8)	17	(6.7)
Completed college or technical training	20	(32.8)	64	(25.4)
Some university (at least 1 year)	10	(16.4)	14	(5.6)
Completed university degree	15	(24.6)	113	(44.8)
Missing	–	–	5	(2)

*ASD* Autism Spectrum Disorder, *DCD* Developmental Coordination Disorder, *PDD* Pervasive Developmental Disorder, *SUO* Service Utilization and Outcomes

### Data analyses

#### Reliability

The reliability assessment was conducted with the proposition that the underlying structure of the AMC-19 is unidimensional. ‘Concern’ as a binary variable and impact as an ordinal variable can show discriminant differences for test re-test and internal consistency [14, 15]. Internal consistency was tested using Cronbach’s alpha in both the AMC-19 Pilot and SUO study. Each study provides a Cronbach alpha score on the concern and impact components. Test-retest reliability was carried out on the AMC-19 Pilot data. The Intraclass correlation (ICC) coefficient was set to 0.80, with the authors willing to accept values of 0.6 or higher.

#### Assessment of scale properties

To assess scale properties, the relationships between AMC-19 impact scores, primary diagnosis, and parent-reported service needs were explored from the SUO dataset using Spearman’s Rho correlations and Kruskal-Wallis test. We hypothesized a priori that diagnoses that are linked to multiple areas of concern will present higher complexity scores. To assess scale properties, we also predicted a positive correlation between AMC-19 impact scores and increased perceived service needs and service utilization.

#### Parental responses

On the initial mail-out parents were asked two open-ended questions on the AMC-19 Pilot survey, and the responses were compiled in the AMC-19 Pilot study database. The first question was: “Are there other things that worry you?”, followed by the second question: “Please feel free to comment on the content of the survey”.

An open inductive thematic analytic approach was used to explore the two questions based on general grounded theory [16]. Comments were repeatedly read until the content was understood. Next, codes (sometimes multiple codes) were marked in each comment, with the codes representing the core idea(s) of the comment. These codes were grouped into broader concepts and categories with a frequency count to manage how often parents reported them. The comparisons of category codes were made after the analysis to confirm the findings. Discrepancies were discussed and resolved by adopting the category titles that were most reflective of the codes within the category until consensus was achieved on all categories. The thematic analysis process and final categorical themes were presented and discussed to ensure a fault did not occur during coding the categories, and the categories were representative of the original parent comments. No theory was generated because the purpose of establishing the categorical themes was to obtain descriptive insights into parental perceptions regarding the utility of the AMC-19 measure from each of the two questions.

## Results

### AMC-19 pilot study

#### Internal consistency

The internal consistency of the AMC-19 for the concern component, evaluated using Cronbach’s alpha, was satisfactory in both the AMC-19 Pilot Time 1 (*n* = 58) and Time 2 (*n* = 39) with values of α = 0.84 and α = 0.81, respectively. The internal consistency for the impact component was α = 0.80 for Time 1 (*n* = 55) and α = 0.81 (*n* = 42) for Time 2.

#### Test-retest

Of the 45 packages returned for the AMC-19 Pilot study, a total of 39 pairs of complete data were available. Six participants were omitted due to missing data so no complexity score was computed. No imputing or replacement of missing values was conducted on the 39 pairs of complete data. Test-retest correlation coefficients for the AMC-19 included an ICC of 0.83 (*p* < 0.001) for the ‘concerns’ and 0.87 (*p* < 0.001) for the ‘impact’ scores.

### SUO study

#### Internal consistency

The internal consistency using Cronbach’s alpha was satisfactory in the SUO study for concern (α = 0.87, *n* = 186) and impact (α = 0.90, *n* = 183). The individual items with the lowest correlations to the full measure total correlation included hearing (*r* = 0.14), mood (*r* = 0.27), and pain (*r* = 0.20). Cronbach’s alpha would remain stable between values of *r* = 0.85 to 0.87 if any of the concern items were to be deleted.

#### Scale properties: Primary diagnoses, service need, and AMC-19 scores

Table 3 shows AMC-19 impact scores arranged by primary diagnosis using the SUO data. Children classified with ASD-related diagnoses showed the highest median score (*n* = 25, median = 30, interquartile range (IQR) 25 = 17, IQR 75 = 41.5); whereas children using the centre for speech services presented the lowest complexity scores (*n* = 46, median = 4, IQR 25 = 2, IQR 75 = 10). A statistically significant difference was found in AMC-19 impact scores among the majority of primary diagnosis groups as determined by a Kruskal-Wallis test, χ^2^(9) = 66.2, *p* < 0.001. Whitney post-hoc tests with Bonferroni correction showed significant differences among all the diagnostic categories at a *p*-value of < 0.05, with the exception of 1) CP and syndrome, and 2) DD and two or more conditions. This finding indicates that in most cases, the AMC-19 detected that median impact scores vary significantly by clinical diagnosis, and the interquartile distribution supports that complexity scores vary within diagnoses.

**Table 3:** Primary Diagnosis and AMC Score

	ASD-related disorders/PDD	CP	DD	Speech	Syndrome	2 or more Conditions
Sample Size	25	20	48	46	16	15
Missing	1	0	5	17	0	1
25th Quartile	17.0	12.5	8.0	2.0	9.8	8.0
50th Quartile	30.0	22.5	15.5	4.0	24.5	14.0
75th Quartile	41.5	37.8	25.8	10.0	31.5	29.0
K-Wallis Ranking*	145.6	131.3	104.6	50.6	124.8	101.4

*All diagnostic categories significant (*p* < 0.05) except 1) CP and syndrome, and 2) DD and 2 or more conditions

ASD, Autism Spectrum Disorder; CP, Cerebral Palsy; DD Developmental Disabilities; PDD, Pervasive Developmental Disorder

Spearman correlations between AMC-19 concern and SAS scores ranged from *r* = 0.32 to *r* = 0.65, all *p* < 0.001, and the correlations between complexity and SAS scores ranged from *r* = 0.31 to *r* = 0.62, all *p* < 0.001 (Table 4). This finding supports that families with higher complexity scores on average perceive higher need for services.

**Table 4:** SAS Scores and AMC Scores

		SAS Child Score	SAS Family Score	SAS TOTAL Score
AMC Concern	Correlation Coefficient	.61^**^	.32^**^	.65^**^
	Sample Size	179	99	179
AMC Impact	Correlation Coefficient	.62^**^	.31^**^	.61^**^
	Sample Size	194	120	194

**p < 0.001

### Parental comments on the AMC-19 measure

#### Question one: “Are there other things that worry you?”

Forty parents responded to the first question, with many comments having multiple codes identified (Table 5). Three major categories were created in the analysis: 1) functioning, 2) participation and sociability, and 3) life-skills.

**Table 5:** Thematic analysis of parental comments of the AMC-19

Question 1: “Are there other things that worry you?”		
Theme	Frequency	Comments
Functioning	22	Language skills (read, write, speak) including effective communication if cannot speak and more one to one therapy
	19	Motor ability with physical recreation activities, around home, dressing, walking, toilet training, fine motor skills (buttons, zippers)
	3	Cognitive ability (e.g., concentration, problem solving, basic knowledge)
	1	Hearing
	1	Memory
Participation and Sociability	11	Involvement in recreation activities (modified for special needs children)
	9	General school integration (such as completing work and enjoying learning)
	7	Acceptance by peers (present or future worry)
Life-Skills	6	Behavioral control and self-regulation (e.g., control temper tantrums and anger, stop hitting self, be less controlling, self-soothe and calm self, regulate emotions, patience)
	5	Awareness of dangers/safety
	3	Handling transition periods and changes in routine
	2	Independent living (e.g., healthier food choices, money management)

The most common reported areas of *functioning* identified as a concern were language skills (*n* = 22) and motor ability (*n* = 19). The second category included *participation and social elements*. The largest area concerned school sociability indicated by general school integration (*n* = 9) and acceptance by peers (*n* = 7). These two areas were present- or future-worry orientation. The next largest area of concern was parents wanting their children to be more involved in recreation activities or to have activities modified for children with special needs (*n* = 11).

Parents identified a concern about *life-skills*, an area, unlike functioning and participation and social elements, that is not currently in the AMC-19. The codes grouped into this concept focused around improving the ability of the child to integrate better into society by improvements in behavior and adaptions to life situations and transitions.

#### Question two: “Please feel free to comment on the content of the survey”

In the second question, the researchers grouped the responses into aspects of the AMC-19 that parents liked and those that needed improvement (*n* = 15; Table 6). Three key themes emerged. The *first* was the expression of concerns. Some parents were already aware of their child’s issues, so the measure did not provide new information to them. On the other hand, parents commented that the AMC-19 is a beginning point to start understanding what families with children with disabilities go through on a daily basis. Some additional comments stated that the AMC-19 allowed parents to reflect on their situation and express concern. The *second* theme identified is the content area covered by the AMC-19. A few parents reported that the measure is vague and did not identify specific areas. In contrast, other parents commented that a positive aspect of the AMC-19 was that the most relevant areas are explored, and parents are able to give detail and suggestions to improve change in therapy. The *third* theme of the AMC-19 involved parents questioning how the AMC-19 will help with planning services. Parents commented that they favor anything that makes services better and makes their “lives a little less challenging”, so they see the AMC-19 as beneficial. A parent commented that the AMC-19 measure is “useful in that it’s more ‘parent-perspective’ than a checklist of yes and no can-do items”.

**Table 6:** Parental evaluation of positive and negative feedback of the AMC-19

Question 2: “Please feel free to comment on the content of the survey”	
*Positive Feedback*	
most relevant areas explored and parents able to give detail and suggestions to improve of change	
some parents found it useful for reflecting on their situation	
parents favor anything that makes services better and makes their lives a little less challenging	
beginning point to starting to understand what families with children with disabilities go through on a daily basis	
useful in that it’s more ‘parent-perspective’ than a checklist of yes and no can-do items	
allows for expressing concern that even if child can-do or may not be good at (especially in the community) so improvements can be made	
*Negative Feedback*	
parents already aware	
parents question how this will help with planning services	
questionnaire vague does not get specific areas	

## Discussion

### Psychometric properties of the AMC-19 measure

This study presents evidence that complexity as assessed by the AMC-19 is a psychometrically sound construct that can be used to initiate dialogue and set goals for children in therapy by identifying the depth of parental concerns in specific areas of daily living. The internal consistency and test-retest scores support that the AMC-19 is a reliable measure.

The high internal consistency scores indicate that the 19-items used in the AMC measure are all necessary and make a meaningful contribution to the measure. Therefore, no items were considered for removal. In a future revision of the AMC-19, items could be added to address parent feedback in regard to general school integration, dangers in environment, handling transition periods and changes in routine, and independent living as suggested by the parents. Should that be done, further psychometric work will be required.

### Relationship between diagnosis and complexity

Complexity scores obtained from the AMC-19 varied both within and between diagnostic categories. While the descriptive data show there is variability among AMC-19 scores by diagnosis, this is not to suggest that one condition is more complex than another. Rather, the intention is to demonstrate that the AMC-19 discriminates levels of complexity both between and within diagnoses. Descriptively, we also found variability among the complexity scores within each diagnosis. The range of complexity scores within the diagnostic categories supports the a priori hypothesis that complexity is a unique construct that differs from child to child regardless of diagnosis. These findings support the longstanding notion of the importance of a ‘non-categorical’ approach to childhood disability [17]. In summary, our findings are supportive of the underlying idea that complexity (concern and impact) is fundamentally different from diagnostic category and thus needs to be measured and treated as such.

### Applications of parental comments: Clinical utility of the AMC-19 and implications for practice

Parents’ comments represented typical concerns of any parent raising a child, with or without a disability. The majority of comments related to areas of functioning already addressed by the AMC-19 measure with the exception of *life-skills*, which is currently not in the AMC-19 but was identified by parents as an area of concern. Life-skills is an important area to explore because of its emphasis on daily independent living that is not socially or functionally focused. Life-skills encompass tasks and situations that may enhance the quality of living by raising awareness in children to prevent children from entering vulnerable situations as well as increasing their confidence to handle difficult situations. On the other hand, one might argue that the need for help with ‘life skills’ reflects the *impact* of the ‘basic’ elements of functional challenge identified by AMC-19, and while very important, should be considered a separate aspect of the lives of young people with developmental challenges.

Many of the concerns identified in Question One were out of the parents’ control – for example, enjoying school or acceptance by peers. Nonetheless, the AMC-19 identifies and voices concerns of parents, so problem areas are prioritized and plans can be created to best mitigate parental concerns. Even if a child can do a task or behavior well, improvements in a task can be monitored based on the areas identified as important by parents. While the breadth of the AMC-19 survey was identified as a limitation, it is necessary in order to capture the most common concerns across multiple areas because specific items will only lengthen the survey, causing burden on parents. Given its broad scope, and used in a clinical setting, the AMC-19 can be helpful to open a dialogue between the therapists/physicians and parents. The administration of the AMC-19 may assist with developing rapport between the therapist and families and also systematically identify key goals of therapy from the families’ perspective. AMC-19 also assists in making parents aware that any or all of the items may be important to them, and allows for the prompt and adequate identification of goals that are important to the family. Overall, the parents’ comments indicate that the use of the AMC-19 in therapy is beneficial to the family-health practitioner relationship.

This study is the first step in assessing the importance that the AMC-19 can have within a clinical setting, and parental perception of the content of the survey was a simple form of face validity. The parents’ comments support that the AMC-19 explores how well the child functions regardless of diagnosis and severity. Parental responses indicate that the AMC-19 measure would have utility in clinical therapy settings by exploring individual functional limitations in the home, school, and community. We suggest therapists provide the AMC-19 measure to parents at the start of therapy or when functional goals are difficult to identify.

### Limitations

The main limitation of both studies is that data were collected from one CTC for the SUO study and three CTCs for the AMC-19 Pilot study, so the range of impairments may have been constrained in some way. We did not sample for a specific number of children with each diagnosis, so the results of the complexities scores are a reflection of the families/parents that responded and were willing to participate in the studies. For example, families from the SUO study reflect a sample with high income and young children. However, respondents with a higher socioeconomic status tend to respond more often to voluntary surveys [18], and children of younger age groups show higher proportions of utilization at CTCs [19–22]. To mitigate issues of representativeness, only descriptive statistics and median rankings were appropriate to analyze on the primary diagnoses and AMC-19 scores, and while we believe the study has internal validity, we caution that no generalizable conclusions about the population have been or should be drawn. Nor do we make implications to infer that specific diagnoses equate to higher or lower complexity levels. In this measurement development study, our goal is not to generalize or draw inferences about the clinical diagnostic population but rather to explore the properties of the AMC-19 as a tool for use between health professionals and families. Nonetheless, the most common disabilities typically seen in CTCs have been captured, which demonstrates the AMC-19 is applicable and appropriate in several settings and populations.

A possible limitation of the AMC-19 Pilot study was a low initial participation rate. This study was not intended to be a rigorous experimental design requiring a large or predetermined sample size, so convenience and respect for the families’ time were the priorities when we selected the sampling strategy. Rather, in this paper, we make a case that complexity is a fundamentally different notion than diagnostic category. Further studies are required to assess the adaptability and utility of the AMC-19 measure in other settings and populations by using a larger sample size.

### Future research directions

Our next step is to assess the validity and clinical utility of the AMC-19 through a larger study by piloting its administration at a CTC. Further research is needed in a population with representation of children with varied diagnoses, chronic impairments or special health care needs. A larger study will evaluate the relationship between parent health and child’s complexity for adequate exploration of a wide variety of children with different conditions and ages, as well as a host of other dependent variables like parental stress as a correlate of the AMC-19 scores. These relationships have implications for understanding family demographics, parents’ well-being, and organizing service allocation.

Currently, we propose using the AMC-19 primarily as a descriptive account of individual children’s complexity. If the AMC-19 is to be used as a comparative instrument, or as a screening or assessment tool, a larger study will inform creating cut-off scores to establish meaningful ‘levels’ of complexity. What is also not yet known is how much real change in AMC-19 scores might be observed over time as children’s development, and their issues, change. Because our samples did not consist of a minimal number of children within specified demographic or other factors, we were unable to determine the amount of invariance in the AMC-19. A more sophisticated research design is required to assess non-invariance in the AMC-19 to provide an adequate conclusion. The implementation of the AMC-19 at a CTC will assess the measure’s utility for identifying priority areas for therapy within a clinical setting. Discussions will make it possible to assess how families and service providers will be involved to inform changes as well as future use of the AMC-19 measure in practice.

## Conclusion

Complexity refers to the aggregation of concerns about children’s health, and the impact of those concerns. The AMC-19 is an instrument designed to identify the complexity in a child’s life to due biological, psychological, social, and environmental issues. The measure is short, easy-to-use, acceptable to families, and has good psychometric properties to explore areas of functional concerns and the impact of different areas on the child from the parental perspective. We believe that AMC provides a structured opportunity for parents to report their perceptions and concerns about their child, and as such offers a view that may be complementary to the way that other observers (e.g., clinicians) assess and evaluate a child’s situation. This family-centred instrument can be used for initiating dialogue between therapists and families, systematically setting goals, and evaluating the impact of supports of children and families in need of additional services.

## Abbreviations

AMC: About My Child
ASD: Autism Spectrum Disorder
CHIEF: Craig Hospital Inventory of Environmental Factors
CP: Cerebral Palsy
CTCs: Children’s Treatment Centres
DCD: Developmental Coordination Disorder
ICF: International Classification of Functioning, Disability and Health
IQR: Interquartile Range
PDD: Pervasive Developmental Disorder
SAS: Supports and Services questionnaire
SUO: Service Utilization and Outcomes study
